# Adherence to Antiretroviral Therapy by Medication Possession Ratio and Virological Suppression among Adolescents and Young Adults Living with HIV in Dar es Salaam, Tanzania

**DOI:** 10.3390/tropicalmed7040052

**Published:** 2022-03-24

**Authors:** Maryam Amour, Raphael Z. Sangeda, Benson Kidenya, Emmanuel Balandya, Blandina T. Mmbaga, Lameck Machumi, Angelica Rugarabamu, Eric Aris, Belinda J. Njiro, Harrieth P. Ndumwa, Eligius Lyamuya, Bruno F. Sunguya

**Affiliations:** 1 Department of Community Health, Muhimbili University of Health and Allied Sciences, Dar es Salaam P.O. Box 65001, Tanzania; sunguya@gmail.com; 2 Department of Pharmaceutical Microbiology, Muhimbili University of Health and Allied Sciences, Dar es Salaam P.O. Box 65001, Tanzania; sangeda@gmail.com; 3 Catholic University of Health and Allied Sciences, Bugando, Mwanza P.O. Box 1464, Tanzania; benkidenya@gmail.com; 4 Department of Physiology, Muhimbili University of Health and Allied Sciences, Dar es Salaam P.O. Box 65001, Tanzania; ebalandya@yahoo.com; 5 Kilimanjaro Christian Medical University College, Moshi P.O. Box 3010, Tanzania; blaymt@gmail.com; 6 Kilimanjaro Clinical Research Institute, Moshi P.O. Box 2236, Tanzania; 7 Management and Development for Health, Dar es Salaam P.O. Box 79810, Tanzania; lmachumi@mdh.or.tz (L.M.); ajrugarabamu@gmail.com (A.R.); aeric@mdh.or.tz (E.A.); 8 Muhimbili University of Health and Allied Sciences, Dar es Salaam P.O. Box 65001, Tanzania; bellyjacky25@gmail.com (B.J.N.); harrieth.peter@gmail.com (H.P.N.); 9 Department of Microbiology and Immunology, Muhimbili University of Health and Allied Sciences, Dar es Salaam P.O. Box 65001, Tanzania; eligius_lyamuya@yahoo.com

**Keywords:** medication adherence, adolescent, young adult, viral load, acquired immunodeficiency syndrome

## Abstract

Background: Adherence to antiretroviral therapy (ART) is a strong determinant of virological suppression. We aimed to determine the magnitude of adherence as measured by medication possession ratio (MPR) and virological suppression with its predictors among adolescents and young adults (AYA) living with HIV on ART in Tanzania. Methods: This retrospective cohort study was conducted using archived data from HIV care and treatment centers in Dar es Salaam, Tanzania between 2015 and 2019. The logistic regression model assessed predictors for adherence and virological suppression. Results: Data of 5750 AYA living with HIV were analysed. The majority were females: 4748 (82.6%). About 63% had good adherence with MPR ≥ 85% at one year post ART initiation. Independent predictors of ART adherence were male sex (aOR = 1.3, 95% CI 1.1–1.5), CD4 > 500 cells/mm^3^ (aOR = 0.7, 95% CI: 0.6–0.9), WHO stage III (aOR = 1.6, 95% CI 1.3–1.9), enrollment in 2019 (aOR = 1.5, 95% CI 1.2–1.9), and virological suppression (aOR = 2.0, 95% CI 1.6–2.9). Using an Efavirenz- and a Nevirapine-based combination was associated with reduced odds of ART adherence (aOR = 0.3, 95% CI 0.1–0.8) and (aOR = 0.2, 95% CI 0.1–0.6), respectively. Predictors of virological suppression were MPR ≥ 85% (aOR = 2.0, 95% CI 1.6–2.4); CD4 > 500 cells/mm^3^ (aOR = 2.4, 95% CI 1.7–3.4), and once-daily dosing (aOR = 2.0, 95% CI 1.3–2.5). Conclusion: Adherence to ART among AYA living with HIV is suboptimal. Sex, year of enrollment, ART drug combination used, and immunological status at ART initiation are important predictors of adherence to ART and virological suppression.

## 1. Introduction

Adherence to antiretroviral therapy (ART) is key to ensure virological suppression among people living with HIV and predicts ART outcomes [1,2]. Adolescents and young adults living with HIV on ART have poorer adherence rates and, consequently, a higher likelihood of failure to achieve virological suppression compared with their adult counterparts [3,4]. A poor adherence to ART thus remains one of the major obstacles to achieving virological suppression [1,5]. Poor adherence results in sub-therapeutic plasma ART drug concentrations, leading to resistance of one or more drugs in a given regimen, and with a possibility of cross-resistance to other drugs in the same class [5]. Suboptimal adherence may include missed or late doses, treatment interruptions, and discontinuations as well as sub-therapeutic or partial dosing [5]. Available evidence suggests that poor adherence among adolescents and young adults may be associated with lack of preferred medication formulation, having too many pills, unfavoured frequency of dosing, stigma, drug toxicities and side effects, age, and developmental stage, as well as psychosocial, economic, behavioral, and socio-demographic characteristics of patients [5,6,7]. Owing to their unique set of behavioral characteristics, adolescents and young adults are subjected into a higher risk for low adherence to ART, increasing their risk for drug resistance and morbidity [3].

The desirable levels of adherence with consequent favourable virological outcomes can be achieved in the adolescents and young adults’ population. Adolescents achieving 100% adherence at 12 months are significantly more likely to exhibit virological suppression [3]. Attaining adequate adherence is necessary to delay viral rebound [3]. In 2015, a global initiative for fast track targets was launched aiming to ending the AIDS epidemic by 2030 [8]. With poor HIV indicators among adolescents and young adults, it is critical to accelerate efforts targeting this population. There are several methods available to measure adherence to ART [9].In clinical practice, appropriate adherence measures can be difficult to confirm and may under or overestimate adherence. Owing to its objective nature, this study utilized the medication possession ratio (MPR), defined as the proportion of time where medication supply was available, to estimate adherence of ART in these individuals [9,10].

Evidence on adherence to ART among adolescents and young adults with HIV is limited in Tanzania. This study thus aimed, first, to investigate the medication possession ratio (MPR) as a proxy measure for adherence among adolescents and young adults living with HIV on ART; second, to identify factors associated with good MPR; and third, to explore the relationship between MPR and virological suppression among adolescents and young adults in Tanzania.

## 2. Materials and Methods

### 2.1. Study Design and Setting

This retrospective cohort study was based on secondary data on HIV patients attending care and treatment clinics (CTCs) in Dar es Salaam, Tanzania supported by the Management and Development for Health (MDH). MDH is a Tanzania-based non-governmental organization supporting HIV/AIDS care and treatment services in 116 CTCs in Dar es Salaam, Kagera, Geita, Tabora, Pwani, and Kigoma regions. The program is funded by the President’s Emergency Plan for AIDS Relief (PEPFAR) through the Centers for Disease Control and Prevention (CDC) to provide ART access as well as HIV management and care in Tanzania [11].As for other PEPFAR-supported programs, the MDH reports data on HIV patients directly to the Tanzania Ministry of Health, Community Development, Gender, Elderly, and Children (MOHCDGEC) using electronic CTC-1 forms. The records were retrieved from the CTC 2 database. Dar es Salaam is the largest city and commercial capital in Tanzania, with an approximate population of six million individuals [12], with the overall HIV prevalence of 4.3%, close to the national average of 4.9% [13].

### 2.2. Study Population and Duration

This study included all adolescents aged 10 to 19 and young adults aged 20–24 years with confirmed HIV and on ART. The study covered five years from 1 January 2015 to 31 December 2019, extracting data for all participants recruited in each study year. Each participant was retrospectively followed up for one year from the time of recruitment into the CTC.

### 2.3. Sampling and Sample Size Estimation

Data of patients aged 10 to 24 years who enrolled in CTC in the Dar es Salaam region between January 2015 and December 2019 were extracted from the CTC-2 database. A total of 159,453 visits data corresponding to 13,341 patients’ data were obtained. Of these, 7591 (55.9%) of the records were excluded because of missing information on ART, stopped ART or was transferred to start or continue with ART or lost to follow-up in less than a year after initiation, or on ART in another center that is not MDH supported. All the remaining 5750 patients were included in the final analysis. 

### 2.4. Study Measures

We extracted de-identified baseline data from the CTC-2 electronic-based records with routinely collected HIV data throughout MDH-supported CTCs in Dar es Salaam. Demographic data studied included age; sex; and facility attended, whether private or public. Clinical data assessed were ART regimen combination at baseline (dolutegravir-, nevirapine-, efavirenz-, or protease inhibitor-based), dose, virological suppression (Yes or No), WHO stage (I, II, III, IV), and absolute CD4 count.

### 2.5. Medication Possession Ratio (MPR)

ART adherence was quantified using MPR defined as the proportion of time when medication supply was available to estimate adherence to ART among the participants [9]. The MPR was computed by dividing the number of days to the next appointment since the last visit that ART was dispensed, to that patient’s total follow-up time (i.e., 365 days) since ART initiation multiplied by 100%. MPR thus provided a more objective means of assessing adherence with minimal risk for over- or under-estimation. MPR was reported as a categorical variable, whereby MPR ≥ 85% was termed as good MPR or good adherence and MPR < 85% was defined as poor MPR or poor adherence [14]. The main outcomes of interest were MPR and viral load suppression within one year of ART initiation.

### 2.6. Data Processing and Analysis

The data were analyzed using STATA version 16. Descriptive analyses were conducted to determine differences in characteristics of the study population and stratified by good or poor MPR. A good possession ratio was defined as MPR greater than or equal to 85%. To compare proportions, we used chi-square tests. Continuous data were compared as medians using the Kruskal–Wallis test. Two tailed *p*-values of less than 0.05 were considered statistically significant. To control for missing CD4 data, we used multiple imputations with chained equations to handle the remaining missing data. We used ordinal logistic regression for the categorical CD4 count categories controlling for age, sex, and other baseline covariates in the imputation. In this analysis, ten imputations were conducted [15].We then performed logistic regression to study the association and possible confounders of individual predictors of good MPR 12 months from ART initiation. Predictor variables included in this analysis were age, sex, type of ART combination, CD4 count, viral load, the dosage of ART, and WHO staging.

### 2.7. Association between MPR and Virological Suppression

As per guidelines by the Tanzania National AIDS Control Program, we considered patients to have virological failure if they did not achieve a viral load <1000 copies/mL within six months of initiating ART or if they had a sustained recurrence of viremia to >1000 copies/mL after initial viral suppression [14].We then performed logistic regression to study the predictors and possible confounders of good MPR and viral load suppression while controlling for the aforementioned factors. Variables were included in the multivariate model if they had a *p* < 0.2 in univariate analysis. We selected the final model using a backward elimination procedure and retained all variables in the model that had a *p* < 0.05.

### 2.8. Ethical Approval

Ethical clearance was obtained from the Research and Ethical Committee of the Muhimbili University of Health and Allied Sciences (MUHAS) with Institutional Review board (IRB) number A.282/298/01. Permission to use this data was obtained from the National AIDS Control Program (NACP) Tanzania through the MOHCDGEC and the MDH. 

## 3. Results

A total of 5750 participants were enrolled in the study, with adolescents totaling 1697 (29.5%) and young adults 4053 (70.5%). Of the 5750 participants, 3603 (62.7%) had good adherence with MPR ≥ 85%. The majority of participants (4748, 82.6%) were females and about a third (1734, 30.2%) were enrolled in 2017. The majority of participants (93.1%) were on an efavirenz-based regimen at baseline, taking pills once per day (5167, 89.9%) and in WHO stage I (3830, 66.6%). More than one-third (1224, 38.6%) had a baseline CD4 count more than 500 cells/mm^3^.

**Figure 1:**
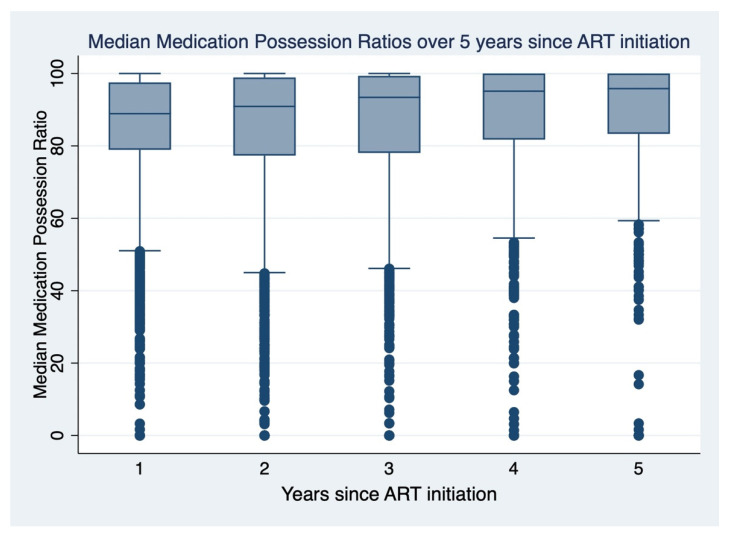
Trends of the median medication possession ratio over the years since ART initiation.

There was a significant association between sex, age, ART regimen combination, year of enrolment, WHO stage at enrollment, and baseline CD4 at enrollment with MPR (Table 1). Trends in median MPR have been significantly increasing over the years from 2015 to 2019 (Figure 1).

Factors associated with good MPR among adolescents and young adults with HIV on ART are summarized in Table 2. In a univariate logistic regression analysis, male sex, being virologically suppressed, and being enrolled in 2018 or 2019 were significantly associated with good MPR. However, having age between 20 and 24 years, baseline CD4 count of >500 cells/mm^3^, having WHO stage II or III, and being on an efavirenz-based regimen were associated with reduced good MPR.

In multivariate analysis, we adjusted for sex, CD4 count, enrollment year, WHO stage, number of pills, viral load, and regimen combination. Factors that remained significantly associated with MPR were sex, WHO stage, CD4 count, virological suppression, and year of enrollment. The odds of having good MPR among males was 30% higher compared with female counterparts (aOR 1.3; 95% CI 1.1–1.5; *p*-value = 0.002). Compared with individuals with WHO stage I at baseline, the odds of having good MPR was 30% more likely for those with WHO stage II (OR 1.3; 95% CI 1.1–1.5; *p*-value < 0.001) and 60% more likely for those with WHO stage III (OR 1.6; 95% CI 1.3–1.9; *p*-value < 0.001). Similarly, having a baseline CD4 count of >500 cells/mm^3^ compared with CD4 count <200 cells/mm^3^ was associated with 30% lower odds of having good MPR (aOR 0.7; 95% CI 0.6–0.9; *p*-value < 0.009). Being enrolled in 2018 was associated with 30% increased odds of good MPR (OR 1.3; 95% 1.1–1.5; *p*-value = 0.017), while being enrolled in 2019 was associated with 50% increased odds of good MPR (OR 1.5; 95% 1.2–1.9; *p*-value = 0.001) compared with 2015. Having virological suppression at one-year post ART was associated with two times the odds of having a good MPR (aOR 2.0; 95% CI 1.6–2.4; *p*-value < 0.001).

Factors associated with viral load suppression among adolescents and young adults with HIV on ART are summarized in Table 3. In univariate logistic regression analysis, having a good MPR, being a female, participants being in the 20–24 age group, having a higher CD4 count, using a once-daily pill, and being enrolled in 2018 and 2019 were associated with an increased chance of achieving viral load suppression. In the multivariate regression model, factors independently associated with viral load suppression were good MPR, higher CD4 count, and once-daily ART dosing. Compared with individuals with a CD4 count <200 cells/mm^3^, those with a CD4 count of 201–349 were 40% more likely to be virologically suppressed (aOR 1.4; 95% CI 1.02–2.0; *p*-value = 0.037), those with a CD4 count of 350–500 cells/mm^3^ were 80% more likely to be virologically suppressed (aOR 1.8; 95% CI 1.2–2.6; *p*-value = 0.005), and those with a CD4 count >500 cells/mm^3^ were 2.4 times more likely to be virologically suppressed (aOR 2.4; 95% CI 1.7–3.4; *p*-value < 0.001). Once-daily dosing compared with twice daily was associated with twice the odds of virological suppression (aOR 2.0; 95% CI 1.3–2.5).

## 4. Discussion

This retrospective cohort study found that 63% of adolescents and young adults had good adherence to ART, defined by MPR above or equal to 85%. Further, we found that male sex, WHO stage II and III, viral load suppression, and being initiated on ART in the years in 2018 and 2019 compared with 2015 were associated with good MPR. Meanwhile, having a CD4 count of more than 500 cells/mm^3^ was associated with 30% lower odds of having good MPR. Nearly 80% of the participants were virologically suppressed. Viral suppression was associated with taking pills once per day, while having good MPR and a higher CD4 count were associated with viral load suppression. 

The level of adherence among adolescents and young adults in this study is not optimal, but it is in keeping with observations made in other studies in this population [2,16,17,18]. In a study conducted in North-Eastern Tanzania, among children and adolescents, 62.5% achieved optimal adherence according to viral load measurements and 65.3% according to pill count [16]. On the contrary, another study in Dar-es-Salaam Tanzania among children 2 to 14 years of age reported adherence assessed via medication return of up to 98% [19]. In this study, 79% of adolescents and young adults were virologically suppressed at one-year post-ART initiation, and thus were a few steps short from achieving the UNAIDS third 90% target [2]. However, the progress is much better in all age groups, with 83% being aware of their status, 90% on treatment, and 92% being virologically suppressed, respectively [8].

Female adolescents and young adults bear the biggest brunt of AIDS in Tanzania. Similar to the global statistics, 75% of adolescents and young adults in the present study were females [20]. In this study, being female was associated with a lower likelihood of having a good adherence. This is in keeping with findings from other studies [2,21]. Female children and adolescents were two times more non-adherent to ART in a study conducted in Northern Tanzania [21]. As MPR is linked to CTC attendance, and because of the nature of women being caretakers of families, it is likely that being preoccupied with household activities may be the plausible reason for non-attendance to CTC and, consequently, low adherence. Given that, the attendance to CTC was an essential variable in computing the MPR in this study. 

In the present study, we observed a negative association between adherence to ART and CD4 count. In both adolescents and young adults, having a CD4 count >500 cells/mm^3^ was associated with lower odds of having good adherence. Similarly, adolescents and young adults with WHO stage I disease were less likely to be adherent to ART compared with those with WHO stage II and stage III disease at baseline, implying that patients who perceive their status as being relatively healthy are less likely to be adherent to ART. This finding is similar to what was observed in other studies including young people with HIV [17,18,22]. We noted that adolescents and young adults with WHO stage III disease were less likely to be virologically suppressed. However, this was not observed among those with WHO stage IV disease and could be a result of discrepancy in subjective WHO clinical staging. Patients with a low CD4 count or advanced WHO stage have reduced immunity and are thus sicker and more prone to opportunistic infections. This could mean that patients who are symptomatic or who perceive a heightened risk of morbidity or mortality are more likely to be adherent to ART in order to improve their health status and get better. In this study, there were proportionally more males with advanced stage disease at ART initiation, and this could have led to the good adherence seen among males as they perceived themselves to be sicker. However, they were less likely to achieve virological suppression. A study in Uganda among children aged 2 to 18 years reported improved adherence among patients who had been hospitalized two or more times [23]. Further, a CD4 count >500 cells/mm^3^ at baseline was associated with more than two-fold higher likelihood of being virologically suppressed at one year post ART initiation. It is noteworthy that being virologically suppressed at one year post ART initiation was associated with good adherence to ART or good MPR.

In the present study, there was a significant improvement of adherence to ART at one year among those initiated on therapy in 2018 and 2019 compared with 2015. Those initiated recently are probably more adherent and it could be that pre-ART counseling is better performed now than it was before. Additionally, longitudinal data over five years suggest an increase in median MPR from 2015 to 2019. This could mean that, as the patient is exposed to CTC for a longer duration, they get used to the experience, improving their adherence to ART. Similar to other studies [24,25] we found that once-daily dosing compared with twice-daily dosing was associated with two times higher odds of being virologically suppressed. Most of the once-daily regimens are relatively newer and more potent with fewer side effects and less resistance, likely contributing to the improved virological suppression observed.

This study has significant limitations owing to its retrospective nature and we had no control of specific variables needed. Therefore, we were not able to ascertain the mode of infection, whether perinatal or horizontal, and disclosure of HIV status as these may affect the adherence to ART, especially among older adolescents and young adults. Additionally, owing to the study’s retrospective nature, we encountered missing data, especially on the CD4 count and viral load. This study used the MPR as a proxy for medication adherence as these patients receive ART during the CTC visit. The strength of this method is that it is more objective than self-reported adherence, as that may lead to overestimating of adherence. However, the MPR has its own limitation in that it can only be assessed based on the ART dispensing record and we cannot prove that the patient indeed consumed the ART dispensed. The strength of this study also relies on the fact that it involved five years’ worth of a large cohort of over 5000 adolescents and young adults from different CTCs in Dar-es-Salaam, Tanzania. These findings may be generalizable to adolescents and young adults living with HIV on ART in Tanzania and other countries in sub-Saharan Africa.

## 5. Conclusions

Only two in three adolescents and young adults had optimum adherence to ART. Predictors of adherence differ from predictors of virological suppression among adolescents and young adults living with HIV and on ART. Factors associated with good adherence to ART were male sex, low CD4 count, WHO stage II and III, viral load suppression, and ART initiation in 2018 and 2019 compared with 2015. Moreover, factors associated with viral load suppression were good medication possession ratio, higher CD4 count, and being on a once-daily pill. Sex and immunological status at ART initiation are important predictors of adherence to ART and virological suppression thereof. We recommend prospective studies to better understand the potential roles of these factors in predicting adherence in this age group.

## Figures and Tables

**Table 1:** Demographic and clinical characteristics of the study participants.

		MPR	MPR	
Variable	Total (*n* = 5750)	≥85% (*n* = 3603)	<85% (*n* = 2147)	*p*-Value
Sex				
Male	1002	698 (69.7)	304 (30.3)	
Female	4748	2905 (61.2)	1843 (38.8)	<0.001
Age categories				
10–19	1697	1102 (64.9)	595 (35.1)	0.021
20–24	4053	2501 (61.7)	1552 (38.3)	
Year enrolled				
2015	705	415 (58.9)	290 (41.1)	
2016	1073	618 (57.6)	455 (42.4)	<0.001
2017	1734	1081 (62.3)	653 (37.7)	
2018	1582	1026 (64.8)	556 (35.2)	
2019	656	463 (70.6)	193 (29.4)	
Regimen Combination				
DTG-based	49	43 (87.8)	6 (12.2)	
Efavirenz-based	5351	3341 (62.4)	2010 (37.6)	0.001
Nevirapine-based	319	196 (61.4)	123 (38.6)	
PI-based	25	18 (72.0)	7 (28.0)	
Other	6	5 (83.3)	1 (16.7)	
WHO Stage				
WHO stage I	3830	2296 (59.9)	1534 (40.1)	
WHO stage II	984	653 (66.4)	331 (33.6)	<0.001
WHO stage III	356	609 (69.8)	264 (30.2)	
WHO stage IV	24	45 (71.4)	18 (28.6)	
Baseline CD4 in cells/mm^3^	(*n* = 3172)			
≤200	570	400 (70.2)	170 (29.8)	
201–349	648	452 (69.7)	196 (30.3)	<0.001
350–500	730	512 (70.1)	218 (29.9)	
500 and above	1224	768 (62.8)	456 (37.2)	
Regimen pills				
Once daily pill	5167	3217 (62.3)	1950 (37.7)	0.060
Twice daily pills	583	386 (66.2)	197 (33.8)	

MPR: medication possession ratio, WHO: World Health Organisation, BMI: body mass index, EFV: efavirenz, DTG: dolutegravir, NVP: nevirapine, PI: protease inhibitor.

**Table 2:** Factors associated with a good (≥85%) medication possession ratio (*n* = 5750).

	Unadjusted		Adjusted	
Variable	OR (95% CI)	*p*-Value	aOR (95% CI)	*p*-Value
Sex				
Female	1.0	<0.001	1.0	
Male	1.5 (1.3–1.7)		1.3 (1.1–1.5)	0.002
Age				
10–19	1.0	0.021	1.0	
20–24	0.9 (0.8–0.97)		0.9 (0.8–1.1)	0.394
CD4 count				
≤200	1.0		1.0	
201–349	0.9 (0.8–1.2)	0.707	0.9 (0.8–1.2)	0.946
350–500	0.9 (0.7–1.1)	0.422	0.9 (0.8–1.1)	0.588
>500	0.7 (0.6–0.9)	0.003	0.7 (0.6–0.9)	0.009
WHO Stage				
WHO stage I	1.0		1.0	
WHO stage II	1.3 (1.1–1.5)	<0.001	1.3 (1.1–1.5)	0.004
WHO stage III	1.5 (1.3–1.8)	<0.001	1.6 (1.3–1.9)	<0.001
WHO stage IV	1.7 (0.9–2.9)	0.068	1.4 (0.8–2.6)	0.214
Regimen Combination				
DTG-based	1.0		1.0	
EFV-based	0.2 (0.1–0.5)	<0.001	0.3 (0.1–0.8)	0.018
NVP-based	0.2 (0.1–0.5)	<0.001	0.2 (0.1–0.6)	0.003
PI-based	0.4 (0.1–1.2)	0.100	0.3 (0.1–1.2)	0.101
Number of pills				
Once a day pills	1.0		1.0	
Twice a day pills	1.2 (0.9–1.4)	0.060	1.3 (0.9–1.8)	0.090
Year Enrolled				
2015	1.0		1.0	
2016	0.9 (0.8–1.2)	0.596	0.9 (0.7–1.1)	0.315
2017	1.2 (0.9–1.4)	0.110	1.1 (0.9–1.4)	0.174
2018	1.3 (1.1–1.5)	0.006	1.3 (1.1–1.5)	0.017
2019	1.7 (1.3–2.1)	<0.001	1.5 (1.2–1.9)	0.001
Virologically				
Not suppressed	1.0		1.0	
Suppressed	1.8 (1.5–2.2)	<0.001	2.0 (1.6–2.4)	<0.001

OR: odds ratio, aOR: adjusted odds ratio, CI: confidence interval, BMI: body mass index, WHO: World Health Organisation, EFV: efavirenz, DTG: dolutegravir, NVP: nevirapine, PI: protease inhibitor, adjusted for all variables in the model.

**Table 3:** Factors associated with virological suppression (*n* = 5750).

	Unadjusted		Adjusted	
Variable	OR (95% CI)	*p*-Value	aOR (95% CI)	*p*-Value
Sex				
Male	1.0	<0.001	1.0	
Female	1.8 (1.4–2.2)		1.3 (0.9–1.7)	0.122
Age				
10–19	1.0	<0.001	1.0	
20–24	1.7 (1.4–2.0)		1.2 (0.9-1.4)	0.109
CD4 count				
≤200	1.0		1.0	
201-349	1.5 (1.1–2.1)	0.011	1.4 (1.02–2.0)	0.037
350–500	1.9 (1.4–2.7)	0.001	1.8 (1.2–2.6)	0.005
>500	2.5 (1.9–3.3)	<0.001	2.4 (1.7–3.4)	<0.001
WHO Stage				
WHO stage I	1.0		1.0	
WHO stage II	0.7 (0.6–0.9)	0.002	0.9 (0.7–1.1)	0.318
WHO stage III	0.5 (0.4–0.7)	<0.001	0.7 (0.6–0.9)	0.001
WHO stage IV	0.7 (0.4–1.4)	0.358	1.2 (0.6–2.4)	0.689
Regimen Combination				
DTG-based	1.0		1.0	
EFV-based	0.4 (0.1–1.2)	0.101	0.5 (0.5–1.4)	0.081
NVP-based	0.1 (0.05–0.4)	0.001	0.4 (0.1–1.3)	0.127
PI-based	0.1 (0.02–0.4)	0.100	0.3 (0.1–1.2)	0.090
Number of pills				
Twice a day pills	1.0		1.0	
Once a day pill	2.5 (2.0–3.3)	<0.001	2.0 (1.3–2.5)	0.003
Year Enrolled				
2015	1.0			
2016	1.0 (0.8–1.4)	0.853		
2017	1.1 (0.8–1.4)	0.586		
2018	1.4 (1.1–2.0)	0.021		
2019	2.7 (1.9–4.0)	<0.001		
MPR				
MPR < 85	1.0		1.0	
MPR ≥ 85	1.8 (1.4–2.3)	<0.001	2.0 (1.6–2.6)	<0.001

OR: odds ratio; aOR: adjusted odds ratio; CI: confidence interval; BMI: body mass index; WHO: World Health Organisation; EFV: efavirenz; DTG: dolutegravir; NVP: nevirapine; PI: protease inhibitor; adjusted for sex, age, CD4 count regimen combination, WHO stage, and number of pills.

## Data Availability

Data can be made available upon request.
